# Resonant Acoustic Rheometry for Real Time Assessment of Plasma Coagulation in Bleeding Patients

**DOI:** 10.21203/rs.3.rs-4784695/v1

**Published:** 2024-10-16

**Authors:** Cheri Deng, Weiping Li, Connor Bunch, Sufyan Zackariya, Shivani Patel, Hallie Buckner, Shaun Condon, Matthew Walsh, Joseph Miller, Mark Walsh, Timothy Hall, Jionghua (Judy) Jin, Jan Stegemann

**Affiliations:** University of Michigan; University of Michigan; Henry Ford Hospital; Henry Ford Hospital; Henry Ford Hospital; Henry Ford Hospital; Henry Ford Hospital; Equation 1 LLC; Henry Ford Hospital; Saint Joseph Regional Medical Center; University of Michigan; University of Michigan; University of Michigan

**Keywords:** Plasma coagulation, Hemorrhage, Viscoelasticity, Thromboelastography, Surface waves, Resonant Acoustic Rheometry, Transfusion

## Abstract

Disordered hemostasis associated with life-threatening hemorrhage commonly afflicts patients in the emergency room, critical care unit, and perioperative settings. Rapid and sensitive hemostasis phenotyping is needed to guide administration of blood components and hemostatic adjuncts to reverse aberrant coagulofibrinolysis. Here, resonant acoustic rheometry (RAR), a technique that quantifies the viscoelastic properties of soft biomaterials, was applied to assess plasma coagulation in a cohort of bleeding patients with concomitant clinical coagulation assays and whole blood thromboelastography (TEG) as part of their routine care. RAR captured the dynamic characteristics of plasma coagulation that were coagulation activators-dependent. RAR coagulation parameters correlated with TEG reaction time and TEG functional fibrinogen, especially when stratified by comorbidities. A quadratic classifier trained on RAR parameters predicted transfusion of fresh frozen plasma and cryoprecipitate with high overall accuracy. These results demonstrate the potential of RAR as a bedside hemostasis assessment to guide transfusion in bleeding patients.

## INTRODUCTION

Disordered hemostasis associated with life-threatening hemorrhage, i.e., hemorrhagic blood failure ^1^, commonly afflicts patients in the emergency room, critical care unit, peripartum, and perioperative settings ^2^. Excessive bleeding is a leading cause of preventable death in trauma and surgical patients ^3, 4^. However, current hemostasis assays are limited in their ability to provide timely hemostasis assessment that clinicians need to make decisions about treatment in resuscitation of severely hemorrhaging patients. In particular, there is an unmet need for rapid and sensitive quantification of blood fibrinogen content and fibrinolytic activity in patients with severe blood loss to guide the administration of blood components and hemostatic adjuncts to reverse aberrant coagulofibrinolysis^5, 6^.

Currently, clinicians rely on the von Clauss fibrinogen assay to determine fibrinogen levels in plasma^7^, but the assay takes up to two hours at many institutions and is too slow to provide actionable results for the exsanguinating patient. Also, independent of the fibrinogen count, many pathologies (e.g., severe acidemia) impair fibrinogen cleavage and fibrin crosslinking ^8^. Thus functional whole blood assays such as viscoelastic hemostatic assays (VHAs), e.g., thromboelastography (TEG) or rotational thromboelastometry (ROTEM), have been used by medical and surgical specialists as a global hemostatic assessment for hemorrhaging patients ^9, 10^ to adjudicate blood component ratios to the individual patient’s hemostatic phenotype ^11, 12, 13, 14^. VHAs are based on the changing viscoelastic properties during blood clot formation triggered by specially designed coagulation activation reagent mixtures. Studies have shown favorable mortality outcomes and conservation of blood products in VHA-guided trauma resuscitation and massive transfusion across non-traumatic patient populations ^9, 15^. However, clot termination often becomes deranged in hemorrhagic blood failure; hemorrhagic shock can manifest as hyperfibrinolysis, which leads to poor prognosis ^6, 16, 17, 18^. Fibrinolytic potential is dampened in plasma due to the relatively high concentrations of plasminogen activator-inhibitor-1 (PAI-1) because of some platelet activation in the centrifugation process ^19, 20, 21^. Yet current VHAs lack sufficient sensitivity to detect clinically significant fibrinolysis effectively^22^ to guide targeted antifibrinolytic therapy. The empiric administration of antifibrinolytic tranexamic acid (TXA) to hemorrhaging trauma patients appeared to reduce mortality in the CRASH-2 trial, yet empiric TXA use has also been associated with increased thromboembolic events ^23, 24^. Traumatologists are therefore seeking refined, rapid, and sensitive bedside tools to assess the individual patient’s fibrinolytic phenotype to selectively guide TXA ^25, 26^. VHAs may offer the traumatologist a rapid method for deciding which patients need fibrinogen repletion beyond the sparse amount of fibrinogen present in fresh frozen plasma (FFP) (~ 1–3 mg/dL). Yet massive transfusion is ongoing in the resuscitation bay with empiric fixed ratio 1:1:1 of packed red blood cells, FFP, and platelets, the traumatologist may perform serial VHAs to depart from a fixed ratio strategy and tailor the blood component therapy ratios to individual patient’s hemostatic derangement to improve outcomes and conservation of blood products particularly in trauma^9, 15^. However, VHA-guided blood component therapeutic approaches are still applied throughout the hospital setting without an effective means to guide a personalized treatment approach ^2, 18, 27^. Furthermore, existing commercially available VHAs are costly as they require expensive reagents, limited in throughput, and are not readily available in many institutions and clinical settings. These underscore the significant need to develop a cost-effect hemostasis assay for rapid and sensitive hemostasis assays for precision hemorrhagic resuscitation ^16, 26^.

Resonant acoustic rheometry (RAR) is an ultrasound-based technique that we have developed for measuring the viscoelastic properties of soft biomaterials ^28, 29^. The rapid measurement speed and non-contact nature of RAR make the technique particularly suitable for longitudinal characterization of dynamically evolving materials in sterile condition. RAR viscoelastic characterization of gelling hydrogels, such as thrombin-induced fibrin gels and ultraviolet (UV) initiated crosslinking of PEG-norbornene hydrogels, have been validated in our previous study against conventional shear rheometry ^28, 29^. It has also been shown in our previous studies that RAR detected abnormal coagulation of hemophilia A plasma ^30^ and plasma from patients treated with the anticoagulant warfarin^31^.

With the goal to develop RAR as a rapid and sensitive hemostasis assay to guide resuscitation of severely bleeding patients, this retrospective study was designed to test RAR for quantifying plasma coagulation in a cohort of bleeding patients. First, RAR measurements were compared against concomitant clinical tests including Clauss assay (supraphysiologic concentration of thrombin), TEG Citrated Kaolin (CK) (with kaolin as activator) and Citrated RapidTEG^™^ (CRT) assays (with kaolin and Tissue Factor [TF] as activators). Then a quadratic classifier (QC) was trained on RAR data to assess the capability of RAR for predicting transfusion requirements in the cohort of bleeding patients.

## RESULTS

### Patient Demographics, Clinical Test Results, and Transfusion Requirements

Initially, 196 plasma samples from patients were screened after routine Clauss fibrinogen measurement but 152 were excluded due to lack of whole blood TEG performed within one hour of the Clauss fibrinogen measurement. Eventually, plasma samples from 38 patients were included in this study (Table 1). Among them, 26.3% were females (n = 10), and the majority were Caucasian (n = 22, 57.9%) and African American (n = 9, 23.7%). Patients were admitted for major surgery, such as cardiac surgery (n = 5, 13.2%), general surgery (n = 5, 13.2%), and organ transplantation (n = 7, 18.4%). Others were admitted for traumatic injury (n = 5, 13.2%), decompensated cirrhosis (n = 10, 26.3%), septic shock (n = 1, 2.6%), post-cardiac arrest syndrome (n = 1, 2.6%), cardiogenic shock requiring peripheral venoarterial extracorporeal membrane oxygenation (n = 2, 5.3%), and non-cirrhotic gastrointestinal bleeding (n = 2, 5.3%). About 25% of the patients were prescribed an anti-thrombotic agent before hospital admission. A third of the patients expired while hospitalized (n = 14, 36.8%). Co-morbidities included hypertension (n = 22, 57.9%), cirrhosis (n = 13, 34.2%), chronic or end-stage kidney disease (n = 13, 34.2%), heart failure (n = 9, 23.7%), diabetes mellitus (n = 9, 23.7%), coronary artery disease (n = 6, 15.7%), and active malignancy (n = 2, 5.3%).

**Figure 1:**
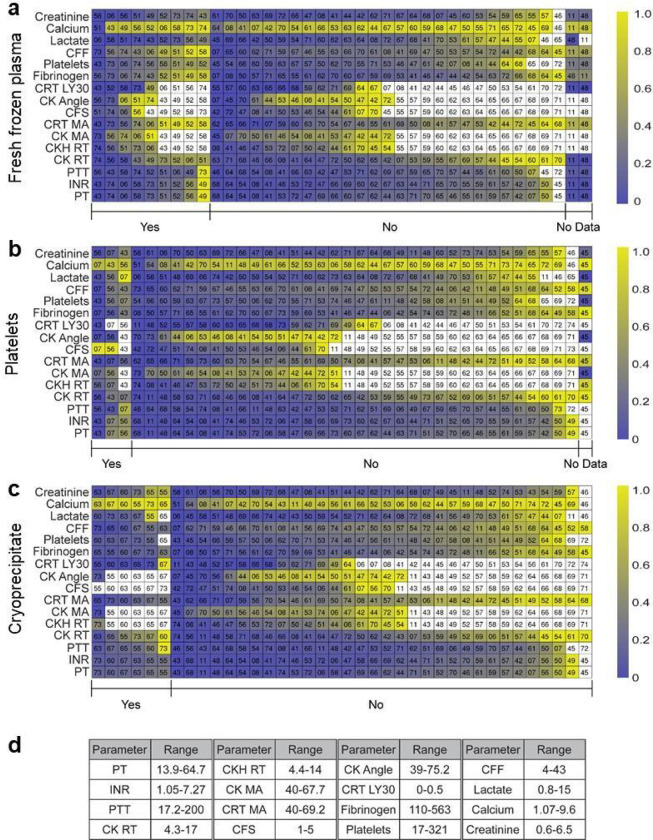
Maps of patients in the cohort arranged based on clinical test results and transfusion decision of fresh frozen plasma **(a)**, platelets **(b)**, and cryoprecipitate **(c)**, respectively. The number in each cell is the numerical identifier for a plasma sample/patient. The groups marked by “yes” represent patients who received a particular blood component, the groups marked by “no” are those without, while the groups marked by “no data” are patients for whom no transfusion data were available. **(d)** Ranges of the clinical test results in this study. PT, prothrombin time (s); INR, international normalized ratio; PTT, partial thromboplastin time (s); CFF, citrated functional fibrinogen (mm); CFS, clot formation speed (min); CK, citrated kaolin; CRT, citrated RapidTEG (min); CK angle; CRT LY30, lysis at 30 min (%); fibrinogen (mg/dL); MA, maximum amplitude (mm); RT, reaction time (min); lactate (mmol/L); Calcium (mg/dL); creatinine (mg/dL).

Concomitant clinical coagulation assays, whole blood TEG^®^ 6s results, and transfusion requirements for the patients in this study are presented as a color-encoded map (Fig. 1), arranged by transfusion requirements of “yes” or “no” for FFP (Fig. 1a), platelets (Fig. 1b), and CRYO (Fig. 1c), as well as the normalized clinical test data against the maximum value among all patients for each specific test (Fig. 1d). Presentation of the clinical data for the cohort of patients in this study in this way clearly shows that none of the transfusion requirements for FFP, platelets, and CRYO could not be delineated by a linear cut-off value of any individual test. These results underscore the notion that clinically, transfusion decision is complex and multifaceted, and could involve many factors such as clinical context, co-morbidities, antithrombotic use, cardiac or renal insufficiency, etc.

### RAR Detected Changes in Viscoelastic Property Associated with Plasma Coagulation

**Figure 2:**
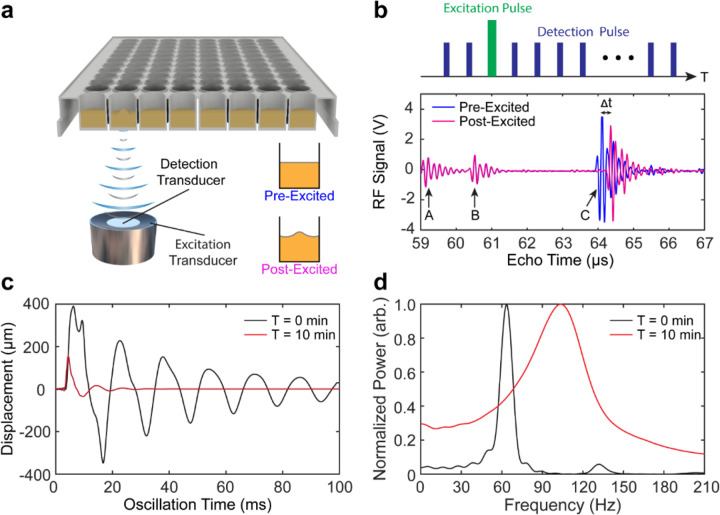
(**a**) Schematic diagram of the Resonant Acoustic Rheometry (RAR) setup using two co-linearly aligned focused ultrasound transducers aiming at the surface of a plasma sample housed in a well of a 96-well microplate to generate and detect the surface deformation or resonant surface waves in the sample. The insets illustrate the sample surface pre- and post-excitation by the “excitation” ultrasound pulse. (**b**) Top: the temporal sequence of excitation ultrasound pulse (green) interleaved with a series of detection pulses (blue) as a function of the elapsed time (T) in RAR measurement for generating and detecting the sample surface movement. The plots show examples of the backscattered signals of the detection pulses from the bottom surface (A) and the top surface (B) of the microplate respectively, as well as the sample surface before (C, blue) and after (magenta) deformation by the excitation ultrasound pulse. The horizontal axis is the travel time of the detection pulse, referred as the echo time. The surface deformation is obtained based on the temporal shift, Δt, of the backscattered signals from the sample surface. (**c**) Examples of the movement or displacement of the sample surface vs. oscillation time measured using RAR before (T=0min; black line) and after (T=10min; red line) coagulation. (**d**) Normalized power spectra of the surface displacements in c, showing increased resonant frequency value from 60 Hz to 100 Hz and width of the frequency peak due to coagulation of the sample (red; T=10min) compared to before (black; T=0min), respectively.

RAR was employed in this study to assess plasma coagulation. As shown in Fig. 2, RAR exploits the novel concept of using a dual-mode ultrasound technique to excite and measure resonant surface mechanical waves in a sample in real time. Application of a short excitation ultrasound pulse (Fig. 2a, b) aiming at the top surface of the sample housed in a standard 96-well microplate (Fig. 2a) was to generate an upward acoustic radiation force to induce an immediate upward deformation of the sample surface ^32^, which subsequently led to the formation of standing surface waves on the circular surface with a boundary. As the result of the formation of resonant surface wave in the sample, the center of the sample surface moved up and down around the equilibrium level. Using ultrasound pulse-echo detection, RAR measures s(τ), the surface displacement s(τ) at the center of the circular surface at a given oscillation time point τ (Fig. 2b) based on the flight time or echo time t, which is the time taken for the detection ultrasound pulse to travel from the transducer to and back from the displaced sample surface. The surface displacement results in a shift in the flight time of the echo signals as Δt=2c∙s(τ), where c is the speed of sound in the sample ^28, 29^ (Fig. 2b). Thus a series of pulse-echo detection operations at a pulse repetition frequency (PRF) of 5 KHz measures s(τ) as a function of τ (Fig. 2c), which exhibited the characteristic behavior of a damped harmonic oscillator. Intuitively and not surprisingly, as a liquid surface was much easier to deform that a solid, RAR detected s(τ) with much larger amplitude and lower frequency in liquid plasma before coagulation (T=0) than in the coagulated sample (e.g., T=10min) (Fig. 2c) when subjected to the same deformation force from the excitation ultrasound pulse.

Uniquely, the characteristics of surface waves depend on the nature of the restoring forces to surface deformations in the material. Thus the dispersion relation, ω=F(k), where ω is the angular frequency of the wave, fundamentally describes how the phase velocity of a wave, $C(\omega )=\frac{\omega }{k}$, depends on its frequency, wavelength or wavenumber, and the mechanical properties of the material ^33^. For example, capillary waves (CWs) are generated on the surface of a liquid and controlled by surface tension of the fluid. On the other hand, Rayleigh waves (RWs), a special class of shear waves generated on the surface of solids, are governed by the shear modulus of the bulk material. In RAR, by exciting and detecting the resonant surface waves in the finite surface of a sample, the wavenumbers are fixed and the available modes of the standing surface waves, or the resonant surface waves, are determined by the radius of the sample surface or the radius of the well, a, and the boundary condition at the well wall^28, 29^. For example, a free boundary condition may be assumed for liquid plasma but a fixed boundary condition may be appropriate for the sample after clotting, thus the surface waves on a circular surface are described by Bessel function of the first or zero order $J_{1}(kr)$ or $J_{0}(kr)$ respectively, where k is the wavenumber. For a specific resonant mode of surface waves, the wavelength, or equivalently the wavenumber, $k_{\left(1,n\right)}$ or $k_{\left(0,m\right)}$, are fixed and determined by the specific roots of the respective Bessel function.

Therefore, the dispersion equation of the surface waves in RAR is reduced such that the frequency directly reflects the mechanical properties of the materials such as surface tension, viscosity, and elasticity ^28, 29, 33^. In short, samples of different properties generate different types of surfaces waves at different frequencies. Stiffer materials generate surface waves with higher frequencies than softer materials, much like that the drums with stiffer (or tighter) drumheads produce higher pitch sounds. For this study, the choice of standard labware, i.e., the 96-well microplates (a≈0.003m), was made for the convenience of testing samples in small volumes, e.g., 100 μL, and that (1, 1) mode of CW and (0, 2) mode of RW have empirically verified as the dominant modes of surface waves in the sample in liquid and solid phase respectively ^28, 29^.

Importantly, RAR leverages a duality of understanding of the surface movement in the sample, both as a damped harmonic oscillator and the a resonant mode of surface wave, offering a novel means to quantify material properties such as surface tension and bulk rheology from the measured surface displacement. Specifically, the frequency of CWs (in liquids) or RWs (in solids), $f_{CW}=\frac{\omega }{2\pi }=\frac{1}{2\pi }\sqrt{\sigma k_{CW}^{3}/\rho }$, or $f_{RW}=\frac{1}{2\pi }\sqrt{Gk_{RW}^{2}/\rho }$, depends on sample density ρ, surface tension σ or shear modulus G ^33^. For the example in Fig. 2, assuming (1, 1) mode of CW and (0, 2) mode of RW before and after plasma coagulation with a=0.003m and $\rho =1020{\;kgm}^{-3}$, the surface tension $\sigma =0.072{Nm}^{-1}$ was determined for the plasma liquid from the measured frequency of 61 Hz at T=0min, while the shear modulus G=128.5Pa was determined in the coagulated sample from the measured frequency of 105 Hz at T=10min. Here, $k_{CW}a=\alpha_{\left(1,1\right)}=3.83$ and $k_{RW}a=\alpha_{\left(0,2\right)}=5.52$. The higher frequency indicated the emergence of shear modulus in the clotted sample that overtakes the effects of surface tension, above a threshold estimated to be G>16.9Pa, from $1<\frac{f_{RW}}{f_{CW}}=\sqrt{\frac{Gk_{RW}^{2}}{\sigma k_{CW}^{3}}}=\frac{k_{RW}a}{{\left(k_{CW}a\right)}^{3/2}}\sqrt{aG/\sigma }$ for (1, 1) mode of CW and (0, 2) mode of RW before and after coagulation.

Furthermore, based on the damped harmonic oscillator model, the full width of the half-peak of the resonant frequency, Δf, also known as the full width at half maximum (FWHM), is directly related to the damping coefficient (Γ) of the surface displacement as $\Delta f=\frac{\Gamma }{\pi }$. On the other hand, damping coefficient of CW or RW depends on material viscosity η, e.g., $\Gamma_{CW}\approx \frac{2\eta k^{2}}{\rho }$ and $\Gamma_{RW}\approx \frac{0.45\eta k^{2}}{\rho }$, respectively. Thus the widening of the frequency peak measured by RAR indicated increased viscosity in the clotted sample after clotting (Fig. 2d).

Taken together, RAR measurements of resonant surface waves via the surface displacement at the center of the sample surface detected the changes in the viscoelastic properties due to plasma coagulation.

### RAR Detected Dynamic Process of Plasma Coagulation

**Figure 3:**
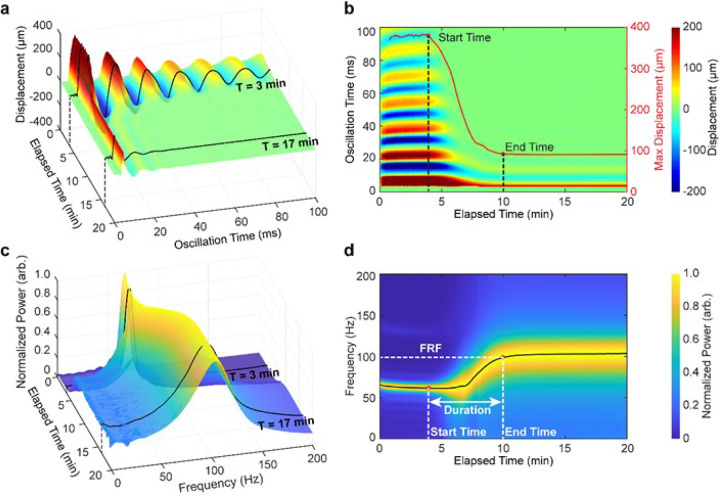
An example of evolving surface displacement and corresponding spectrum during coagulation of a plasma sample using Resonant Acoustic Rheometry (RAR). This RAR result is with addition of 0.05% Tissue Factor as the clotting activator. **(a)** A 3D surface map shows the displacement of the sample surface vs. the elapsed time (T) during coagulation. Black lines highlight the surface displacement at a time before (T=3min) and after notable coagulation (T=17min), respectively. **(b)** The corresponding 2D heatmap of the sample surface displacement shown in **a**. The red line shows the maximum surface displacement (or displacement amplitude) vs. the elapsed time (T), with the vertical dashed line representing the “coagulation Start Time” and “end time” defined based on the change in the displacement amplitude. **(c)**A 3D representation of the power spectrum of the sample surface displacement in **a** vs. T with the black lines indicating the spectrum of the surface displacements at T=3min and 17 min respectively. **(d)** The corresponding 2D representation of the spectrogram in **c**; *i.e*., the power spectrum *vs*. T, showing the RAR Start Time, Duration, End Time, and Final Resonant Frequency (FRF).

Considering that each RAR run included one excitation ultrasound pulse followed by high PRF pulse-echo detection to measure the resonant surface waves that lasted 0.1–0.2 s in the sample, repeated RAR runs at a desired interval, e.g., 5 s or even 1 s, was utilized to capture how the resonant surface waves change over time throughout the process of plasma coagulation. As an example, Fig. 3 and **supplemental audio-video 1** show the RAR measurements of coagulation of plasma from a 64-year-old male (identified as patient #50) with decompensated hepatitis C cirrhosis and a preoperative Model for End-stage Liver Disease (MELD) score of 18. Collectively, the resonant surface waves throughout coagulation over the elapsed time T, presented as a surface plot (Fig. 3a) and especially a color-encoded 2D displacement heatmap (Fig. 3b), clearly show the amplitude of the resonant surface wave, or the maximum surface displacement, which decreased from 380 μm (red line, Fig. 3b) at T=3min (dashed line, Fig. 3b) to less than 95 μm at T=10min. Meanwhile, duration of the surface movement also rapidly reduced from over six cycles of oscillations to just one or two cycles or 20 ms at T=10min (Fig. 3a, b). These decreases are consistent with the expected increase in both stiffness and viscosity of the sample as the results of liquid-to-solid phase transition during coagulation. Correspondingly, the spectrogram (Fig. 3c, d), which is a collection of the frequency spectrum over the elapsed time throughout the process of coagulation, also revealed distinct increases in peak frequency and FWHM during liquid-to-solid transition (Fig. 3c, d), again consistent with increased shear modulus and viscosity in the sample. While RAR readily captured the entire dynamic changes in the resonant surface waves during plasma coagulation in terms of the surface wave amplitude, frequency, and damping coefficient, discrete RAR parameters, Coagulation Start Time, Duration (End Time – Start Time) and Final Resonant Frequency (FRF), are defined (Fig. 3b, d) to quantify the active coagulation process and the strength of the final clot. Here, for easier interpretation and simplicity, Start Time, Duration, and FRF are used rather than the terminology of Liquid Phase Time and Clotting Phase Time used in our previous work which quantified similar characteristics of plasma coagulation ^30^.

The RAR spectrogram exhibited striking similarity with the graphic results of whole blood thromboelastogram (TEG) for this patient (**Figure S1**), which was collected in the pre-anhepatic phase during an orthotopic liver transplantation, suggesting the potential of RAR as a feasible VHA for rapid coagulation assessment.

Taken together, these results show that repeated RAR measurements of the resonant surface waves captured the changes in the resonant surface waves, thus mechanical properties, in a coagulating plasma as the result of phase transition and interplay of surface tension, viscosity, and elasticity in the sample. Since these dynamic changes fundamentally reflect the kinetics of chemical and physical reactions in the material, e.g., polymerization and crosslinking during thrombin-induced fibrin gelation^28^, RAR measurements may offer increased information content and sensitivity compared to conventional rheological approaches.

### Coagulation Characteristics Determined from RAR Assays

**Figure 4:**
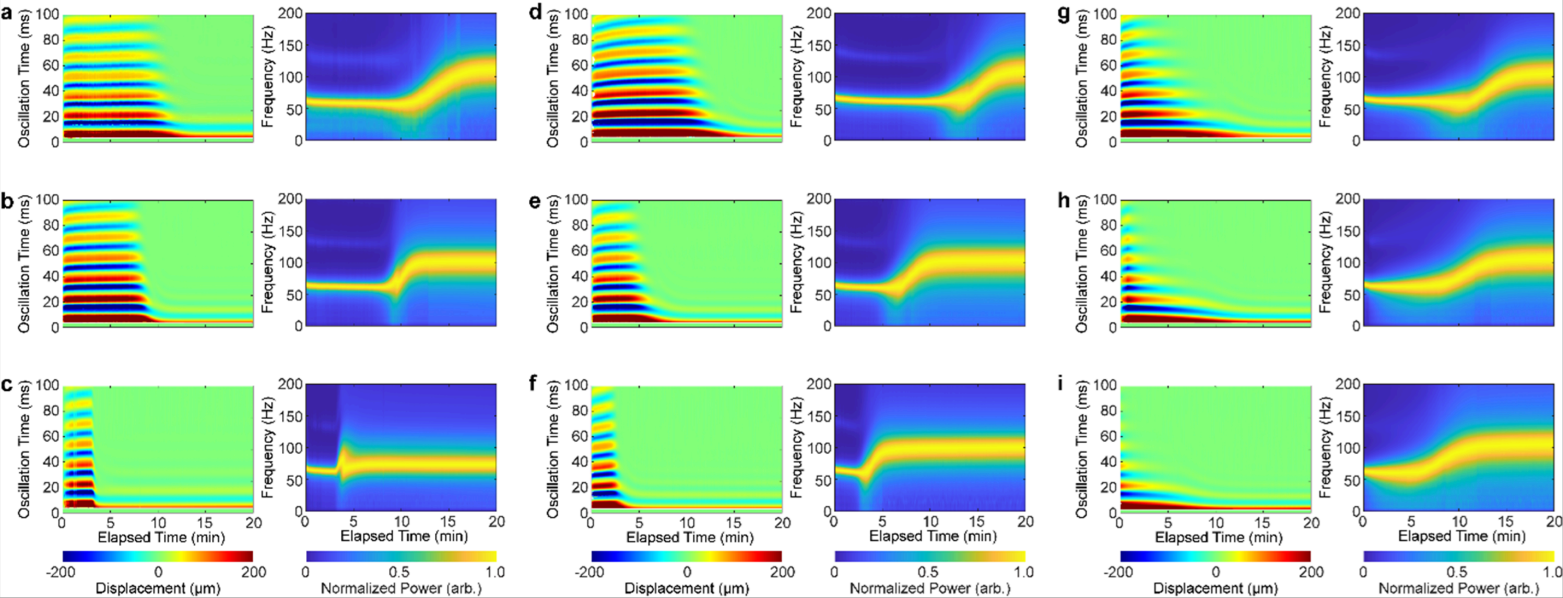
Surface displacement heatmap (left graph) and spectrogram (right graph) measured in a plasma sample using Resonant Acoustic Rheometry (RAR) under the following nine conditions: **(a)** Control (calcium 15 mM, lipids 25 μM), **(b)** CK (calcium 15 mM, lipids 25 μM and kaolin 0.004% w/w), **(c)** CRT (calcium 15 mM, lipids 25 μM, kaolin 2% w/w and TF 2 μg/mL ), **(d)** TF 0.01% (calcium 15 mM, lipids 25 μM and TF 7 μg/mL), **(e)** TF 0.05% (calcium 15mM, lipids 25 μM and TF 35 μg/mL), **(f)** TF 0.2% (Calcium 15 mM, Lipids 25 μM and TF 140 μg/mL), **(g)** Thrombin 0.1 U/mL (Calcium 15mM, Lipids 25 μM and Thrombin 0.1 U/mL), **(h)**Thrombin 0.2 U/mL (Calcium 15 mM, Lipids 25 μM and Thrombin 0.2 U/mL), **(i)** Thrombin 0.4 U/mL (Calcium 15 mM, Lipids 25 μM and Thrombin 0.4 U/mL).

In this study, RAR assays were performed on the plasma sample from each patient in the cohort under nine reagent conditions (Table 2), designed capture and compare coagulation characteristics with concomitant clinical tests including Clauss assay, TEG CK assay, TEG CRT assay, and TEG Tissue Factor (TF) assay. As an example, Fig. 4 shows the results of the nine RAR assays of plasma coagulation for the same patient in Fig. 3, revealing that the coagulation dynamic characteristics are reagent condition dependent. For example, compared to the control reagent condition (reagent condition #1) (Fig. 4a), shortened RAR Start Time and Duration were detected with addition of kaolin in the CK assay (reagent condition #2) (Fig. 4b, **supplemental audio-video 2)** or kaolin and TF in the CRT assay (reagent condition #3) (Fig. 4c), indicated by the rapid decreases in both surface displacement amplitude (left panels, Fig. 4a–i) and increases in frequency (right panels, Fig. 4a–i). Increased concentrations of TF (reagent condition #4–6) (Fig. 4d–f) or thrombin (reagent condition #7–9) (Fig. 4g–i, **supplemental audio-video 3)** also shortened Start Time and Duration.

**Figure 5:**
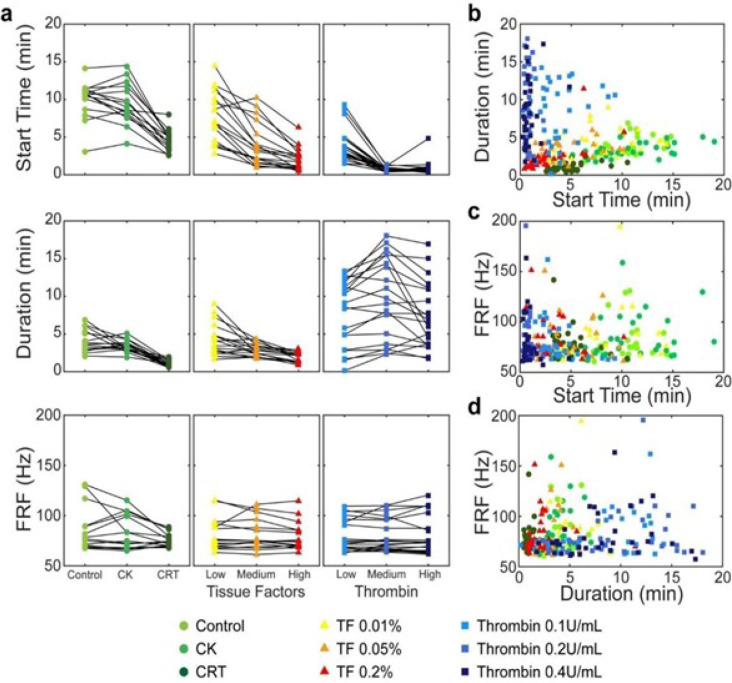
(**a**) Three groups of RAR Start Time, Duration, and Final Resonant Frequency measured under RAR reagent condition #1 – 3 (Control, CK, and CRT), #4 – 6 (TF 0.01%, TF 0.05%, and TF 0.2%), and #7 – 9 (Thrombin 0.1 U/mL, Thrombin 0.2 U/mL, and Thrombin 0.4 U/mL) for all patients in this study. RAR parameters for each patient in each group were connected by a solid line across the three RAR assays in the group. (**b-d**) Scatter plot of Duration vs. Start Time, FRF vs. Start Time, and FRF vs. Duration for all patients across nine RAR reagent conditions.

These trends were observed across all patients (Fig. 5). Compared the results under the control reagent condition, Start Time decreased significantly in RAR assays with kaolin (reagent condition #2 and 3) and TF added as activators (reagent condition #4–6), especially at higher reagent concentrations (top row, left and middle panels, Fig. 5a), while Duration decreased only slightly (middle row, left and middle panel, Fig. 5a). These results indicate the differences in the initiation dynamics between intrinsic and extrinsic coagulation pathways by kaolin and TF respectively. Furthermore, under reagent condition # 7–9, Start Time was significantly shortened in RAR assay with higher thrombin concentrations (top row, right panel, Fig. 5a), while Duration showing only a slight increase (middle row, right panel, Fig. 5a). These results suggest a different mechanism of action of thrombin from kaolin and TF that could be due to the direct effect of thrombin concentration on fibrin polymerization kinetics and the ensuing fibrin fiber thickness^34^. Thrombin triggers the onset of fibrin clot formation, thus, high thrombin concentration will immediately initiate clotting, leading to a much shortened Start Time. On the other hand, in the TF/kaolin assays, increased rate of fibrin generation, fibrin fiber thickness, and fibrin crosslinking could speed the formation of fibrin meshwork. Thus, the TF/kaolin RAR assay may reflect the effect of a “fibrin gradient” with a shorter Duration, which will not occur in the thrombin-activated RAR assays for which only a slightly prolonged Duration was observed.

While general trends existed for Start Time, Duration, and FRF vs. reagent conditions as shown by the clustering of symbols of similar color shades in Fig. 5b–d, lines of the RAR parameters for patients were not all parallel and did not have the same trends across the nine reagent conditions (Fig. 5a). This suggest the differential impacts of coagulation activators in the RAR assays in different patients. However, the sensitivity of RAR to patient demographics and co-morbidities needs to be confirmed in a larger cohort patients.

### Comparison of RAR Parameters with Clinical Test Results

**Figure 6:**
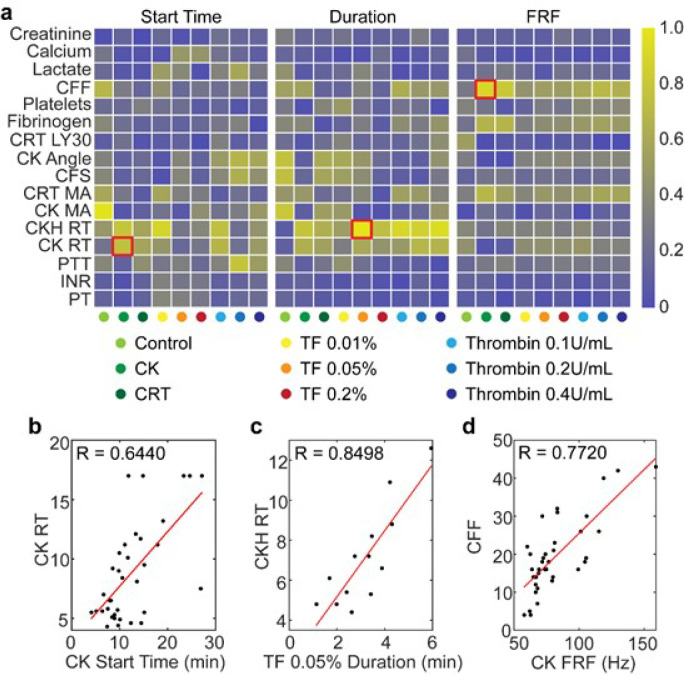
**(a)** Heatmaps of linear correlation coefficient (R) between each of the 16 clinical test results (vertical) and each of RAR Start Time, Duration, and Final Resonant Frequency (FRF) for the nie regent conditions (horizontal) tested in this study. The three red squares were selected examples for correlations of Start Time (left), Duration (middle), and Final Resonant Frequency (FRF) (right) with CK RT, CKH RT, and CFF respectively. **(b-d)** Scatter plot of the data for the selected three examples in **a** showing clinical test results (vertical axis) vs. RAR parameters (horizontal axis). Abbreviations: CK, citrated kaolin; RT, reaction time; CKH, Citrated Kaolin with Heparinase; CFF, citrated functional fibrinogen; CFS, clot formation speed; CRT, citrated RapidTEG; INR, international normalized ratio; LY30, lysis at 30 min; MA, maximum amplitude; PT, prothrombin time; PTT, partial thromboplastin time; RAR, resonant acoustic rheometry; TF, Tissue Factor.

To assess how RAR parameters compare with clinical test results, one to one correlation of each of the RAR parameters with each of the 16 clinical test result was performed (Fig. 6a). As expected, not all RAR reagent condition yielded consistent superior correlation for all RAR parameters with all clinical test results, given that some of the clinical tests were not directly related to the characteristics of plasma coagulation. Nevertheless, it is clear that TEG RT results (CKH RT and CK RT) correlated with Start Time and Duration for most RAR reagent conditions (left and middle panels, Fig. 6a), suggesting that these RAR parameters captured the characteristics associated with enzymatic coagulation initiation by plasma factors and kinetics of fibrin buildup. TEG CFF and Clauss fibrinogen count correlated with RAR FRF (right panel, Fig. 6a). These are further illustrated by selected examples (red squares in Fig. 6a), i.e., Start Time (in CK condition or reagent condition #2) vs. TEG CK RT (R = 0.6440, Fig. 6b), Duration (reagent condition #5 with TF 0.05%) vs. TEG CKH RT (R = 0.8498, Fig. 6c), and FRF (in CK condition or reagent condition #2) vs. TEG CFF (R = 0.7720, Fig. 6d).

Both TEG CFF and Clauss fibrinogen assays demonstrated agreement with RAR FRF, suggesting that RAR may potentially serve as a surrogate for fibrinogen content and fibrinogen activity in plasma without the addition of the glycoprotein IIb/IIIa receptor inhibitor abciximab, which was required in TEG CFF assay to inhibit the platelet contribution to maximal clot strength. Since FRF reflects clot stiffness ^28, 29^, it is not surprising that FRF correlated with TEG CFF and Clauss fibrinogen.

Since none of the 38 patient samples demonstrated hyperfibrinolysis on whole blood TEGs, thus no associations were expected between RAR and TEG LY30. RAR parameters did not correlate with PT/INR/PTT and TEG LY30, as well as creatinine, calcium, lactate, or platelet count. The lack of high individual correlation with PT/INR/PTT, TEG α-angle, CFS, MA, CFF, or LY30 may be because RAR was performed with plasma while TEG with whole blood. Plasma samples lacked erythrocytes contribution to platelet recruitment and final clot contraction strength^35^. As RAR detected changes in surface waves in the sample due to liquid-to-solid transition and material stiffness, it is expected to be more precise than PT/INR/PTT. In addition, RAR was performed on thawed plasma after a period of storage time at freezing temperatures, which could introduce some variability while simultaneously enhancing fibrinogen activity ^36^.

### Correlation of RAR Plasma Results with Clinical Assays Stratified by Co-morbidities

**Figure 7:**
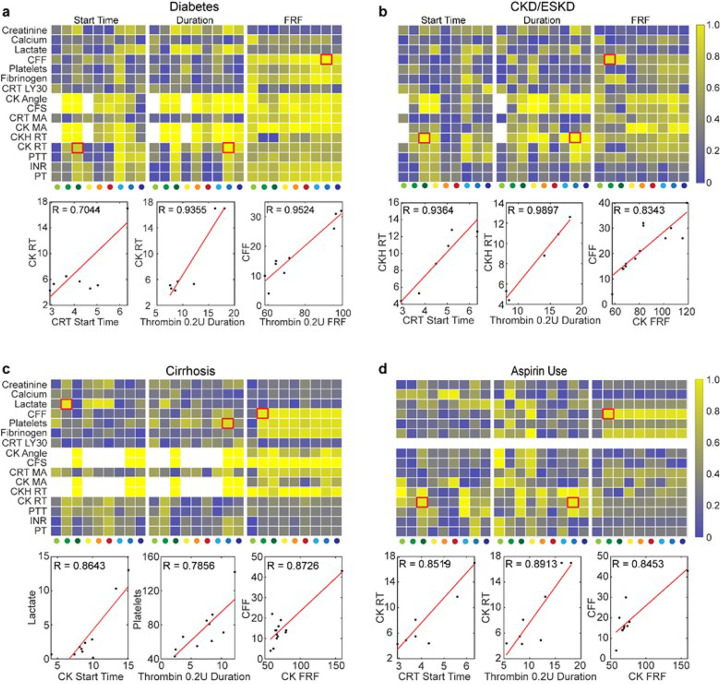
**(a-d)** Linear correlation coefficient (R) heatmaps (top) and representative scatter plots of clinical test results vs. RAR parameters (bottom) for subgroups of patients with diabetes, CKD, cirrhosis, and aspirin use. Abbreviations: CFF, citrated functional fibrinogen; CFS, clot formation speed; CK, citrated kaolin; CKD, chronic kidney disease; CRT, citrated RapidTEG; FRF, final resonance frequency; INR, international normalized ratio; LY30, lysis at 30 min; MA, maximum amplitude; PT, prothrombin time; PTT, partial thromboplastin time; RT, reaction time; TF, Tissue Factor.

When correlations were stratified based on co-morbidities (diabetes mellitus, chronic kidney disease (CKD)/end-stage kidney disease (ESKD), cirrhosis, and aspirin use (Fig. 7), or hypertension and alcohol use (**Figure S2**), stronger correlations of RAR with were obtained for each sub group (bottom panels, Fig. 7a–d) compared to the entire group (Fig. 6b–d), although the sample size for each co-morbidity was small. For diabetes, higher correlation was seen in Start Time (CRT condition or reagent condition #3) vs. CK RT (R = 0.70, bottom left panel, Fig. 7a), Start Time and Duration (CRT and thrombin 0.2 U condition or reagent condition #8) vs. CK RT (R = 0.94, bottom middle panel, Fig. 7a), FRF (Thrombin 0.2U condition or reagent condition #8) vs. CFF (R = 0.95, bottom right panel, Fig. 7a). For patients with CKD/ESKD and aspirin use, higher correlations were also seen (Fig. 7b), including Start Time (CRT condition) vs. CKH RT (R = 0.94, bottom left panel, Fig. 7b), Duration (thrombin 0.2 U condition) vs. CKH RT (R = 0.99, bottom middle panel, Fig. 7b), and FRF (CK condition) vs. TEG CFF (R = 0.83, bottom right panel, Fig. 7b). For cirrhosis and aspirin use patients, Start Time, Duration, and FRF exhibited high correlation with relevant clinical results (Fig. 7c, d).

Strong correlation of FRF with clinical tests for both diabetes and cirrhosis is consistent with the well-documented findings that diabetes produces a subtle and often subclinical coagulopathy, leading to altered fibrin meshwork ^37, 38^. The altered fibrin cross-linkages invoked by uncontrolled elevated blood sugars may explain the strong correlation for FRF to nearly all clinical results. For cirrhotic patients, FRF exhibited strong correlations to clinical test results but not PT/INR/PTT. This may be due to the rebalanced hemostasis caused by the impaired hepatic synthesis of many coagulation factors. It is for this reason that VHAs are now used clinically in liver transplantation and to guide resuscitation of bleeding cirrhotic patients, as PT/INR/PTT have little clinical value in assessing a cirrhotic patient’s rebalanced hemostasis ^39, 40^.

### Prediction of Transfusion Requirements using RAR Parameters

To test whether RAR parameters could predict transfusion requirements, a quadratic classifier (QC) was used for this two-class classification problem to identify true positive (TP) and true negative (TN). Classification was performed for transfusion requirement of FFP and CRYO, but not platelets as only three patients received platelets in this study (Fig. 1b).

**Figure 8:**
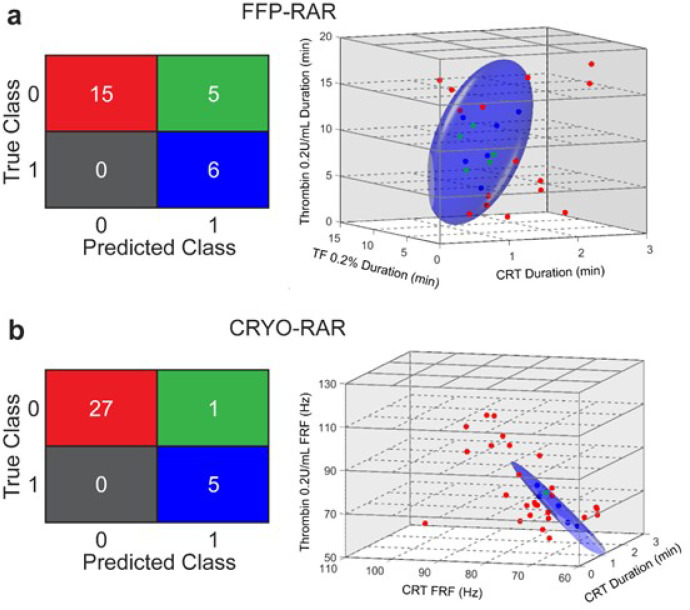
(**a**) Classification confusion matrix and a 3D representation of classification outcome for FFP transfusion requirement using a quadratic classifier (QC) trained on RAR Duration data under the reagent conditions of CRT (reagent condition #3), TF 0.2% (reagent condition # 6), and Thrombin 0.2U/mL (reagent condition #8). The two columns in the confusion matrix represent the true classes of patients who did not receive FFP transfusion (marked as 0) and who did (marked as 1) respectively. The two rows represent the predicted classes by the QC model corresponding to those without FFP transfusion (marked as 0) and with FFP transfusion (marked as 1). The numbers in each of the four cells, 15, 5, 0, and 6 indicate True Negative (TN), False Positive (FP), False Negative (FP), and True Positive (TP), respectively, using the QC model. The colors of the data points in the 3D plot on the right correspond to the colors of the cells in the confusion matrix on the left, separated by the decision boundary determined by QC. (**b**) Confusion matrix and a 3D representation of classification outcomes for CRYO transfusion requirement using a QC trained on RAR Duration from reagent condition CRT (reagent condition #3) and FRF data from reagent conditions CRT (reagent condition #3) and Thrombin 0.2 U (reagent condition #8). The numbers in each of the four cells, 27, 1, 0, and 5 in the confusion matrix indicate TN, FP, FN, and TP, respectively using the QC for CRYO transfusion requirement. The colors of the data points in the 3D plot correspond to the colors of the cells in the confusion matrix, separated by the decision boundary determined by the QC.

Duration and FRF from RAR assay conditions of CRT, TF 0.2%, and thrombin 0.2 U (reagent condition #3, #6, and #8) (Table 2) were used in our classification. After excluding patients with invalid RAR assays (no coagulation was detected during RAR assay) or without transfusion status in the classification, results from 26 patients were used in classification among whom six received FFP (“yes”) and 20 did not (“no”). Classification using a QC predicated FFP transfusion with an overall accuracy of 80.8% (Fig. 8a). The QC correctly predicted all six “yes” cases, i.e. True Positive (TP) = 6, False Negative (FN) = 0, and 15 out of 20 “no” cases, i.e., True Negative (TN) = 15 and False Positive (FP) = 5. The recall(sensitivity)=TP/(TP+FN)=100%, precision=TP/(TP+FP)=52.3%, specificity=TN/(TN+FP)=75%, and negativepredictivevalue=TN/(TN+FN)=100%. F1-score is $\frac{2\times \;\text{precision}\times \;\text{recall}}{\left(\text{precision}+\text{recall}\right)}=69\%$, a satisfactory score given the unbalanced data sets (23% “yes” vs. 77% “no” cases) and higher risk of missing “yes” cases.

Trained on Duration from reagent condition #3 and FRF from reagent condition #6 and reagent condition #8 for a total of 33 patients with five “yes” and 28 “no” cases, the QC predicted CRYO transfusion with an overall accuracy of 97% (Fig. 8b). Here TP = 5 and FN = 0; TN = 27 and FP = 1, yielding a recall = 100%, precision = 83.3%, specificity = 96.4%, and negative predictive value = 100%. The F1 score is 91%, showing an excellent overall performance of the QC.

Further validation of these results is needed in future studies including a larger number of patients. Also, classification was performed assuming the clinical cases of “yes” and “no” transfusion as truth, which is difficult to verify.

### Classification of Transfusion Requirements using Clinical Test Results

A QC was also trained on PTT, Fibrinogen, and CFF results to test prediction of transfusion requirements. The model predicted FPP transfusion with an overall accuracy of 92.3% (**Figure S3a**). Here, TN = 20 and FP = 0; TP = 4 and FN = 2, yielded a precision of 100%, recall (sensitivity) 66.7%, and F1 score 80.0%. Using PT, Fibrinogen, and CFF, the model predicted CRYO infusion with an overall accuracy of 88.9%, TN = 26, FP = 4; and TP = 6 and FN = 0 (**Figure S3b**). The resultant precision is 60%, recall 100%, and F1 score 75%. These results are comparable with classification based on RAR parameters (Fig. 8) and also validate the clinical decision on transfusion requirements in this study.

The recent CRYOSTAT-2 trial was a negative trial for 28-day mortality benefit of empiric administration of three pools of CRYO (equivalent to ~ 6 g fibrinogen) to patients in traumatic hemorrhagic shock requiring massive transfusion ^41^. Admission fibrinogen levels in severe injury is an independent predictor of mortality, and fibrinogen is one of the first coagulation factors to be severely depleted in exsanguination ^42, 43, 44^. Thus, the question remains as to which severely injured patients benefit from fibrinogen replenishment. The answer likely is not empiric administration to all exsanguinating patients in shock, which was the hypothesis tested in the 1,604 patients CRYOSTAT-2 trial, but rather a personalized medicine approach guided by VHAs such as RAR and TEG CFF to identify patients whose resuscitation requires fibrinogen repletion.

## CONCLUSIONS

RAR provided rapid and sensitive profiling of plasma coagulation. RAR parameter FRF correlated strongly with Clauss fibrinogen and TEG CFF, demonstrating the potential of RAR as a bedside VHA for quantifying fibrinogen activity. Classification using a QC trained on RAR parameters achieved high overall accuracies on prediction of FFP and CRYO transfusion requirements. These results lay the groundwork for future studies to develop RAR as a rapid and sensitive hemostasis assay to guide clinical decision-making in hemorrhagic resuscitation.

## METHODS

### Study Design and Sample Collection from Participants

This retrospective observational study was conducted in accordance with the Declaration of Helsinki and the Institutional Review Board on August 30, 2022 (IRB No. 15804). Informed consent was waived as there was no direct patient contact or care involved in the study. Plasma samples were obtained *via* convenience sampling of pathologic plasma samples which had Clauss fibrinogen measured as part of routine clinical care from September 7, 2022, through October 25, 2022. All plasma samples were from non-pregnant adults ≥ 18 years of age. Samples were excluded if 1) not stored in a refrigerator within one hour of collection and not stored in a −20°C freezer within eight hours of collection, 2) contaminated, 3) < 0.3 mL of residual plasma was available after routine clinical use, or 4) a concomitant whole blood TEG assay was not performed within one hour of the time of collection of the plasma sample.

All blood samples were collected in blue top Vacutainer^®^ tubes containing 0.109 M (3.2%) trisodium citrate according to Clinical Laboratory and Standards Institute (CLSI) guideline H03-A6. All TEGs were performed as part of routine clinical care. This study adheres to the reporting guidelines of the Strengthening the Reporting of Observational Studies in Epidemiology (STROBE) statement ^45^. Patient demographics (age, sex, BMI, race), co-morbidities, prehospital antithrombotics, reason for hospital admission, transfusion requirements, and inpatient death were collected. For each patient, whole blood TEG parameters, Clauss fibrinogen count, platelet count, and PT/INR/PTT, were collected and compared with RAR measurements.

### Thromboelastography (TEG)

Using a TEG^®^ 6s device (Haemonetics Corporation, Braintree, Massachusetts, USA), TEG was performed at the Henry Ford Hospital Blood Bank on citrated whole blood after a 10-min incubation period at room temperature following collection, per the manufacturer’s direction. TEG 6s Global Hemostasis^®^ cartridge and Global Hemostasis with Lysis^®^ cartridge were used. The Global Hemostasis^®^ cartridge contains four assay channels, each with unique dried activators/inhibitors: Citrated Kaolin (CK) using kaolin 0.004% w/w and CaCl_2_ 0.9 M; Citrated RapidTEG^™^ (CRT) using kaolin 2% w/w, TF 2 μg/mL, and CaCl_2_ 0.8 M; Citrated Kaolin with Heparinase (CKH) using kaolin 0.007% w/w, CaCl_2_, 0.9 M, and heparinase ≥ 400 IU/mL; and Citrated Functional Fibrinogen (CFF) using TF 0.3 μg/mL CaCl_2_ 0.8 M, and abciximab 2 mg/mL (15). The Global Hemostasis with Lysis^®^ cartridge contains three assay channels including CK, CRT, and CFF. The following quantitative parameters were obtained from the graphic tracing of TEG 6s assay: maximum amplitude (MA) (reference range 52–70 mm), CFF MA (or the MA-FF) (reference range 16–32 mm), reaction time (R, reference range 4.6–9.1 min), α-angle (reference range 63–78°), clot formation speed (CFS, reference range 0.8–2.1 min), and lysis at 30 min (LY30, reference range 0.0–2.6%). An elevated LY30 indicates hyperfibrinolysis, which has a high clinical incidence in the anhepatic phase of liver transplantation, but may also be observed in severe traumatic hemorrhagic shock ^46, 47^.

### Conventional Coagulation Assays, Clauss Fibrinogen, and Platelet Count

PT/INR/PTT and Clauss fibrinogen assays were performed at the Henry Ford Hospital Clinical Chemistries Laboratory using the STA-R Compact Max^®^ analyzer (Diagnostica Stago, Parsippany, New Jersey, USA), per manufacturer’s instruction. Specifically, citrated whole blood samples were centrifuged at 1500 g for 15 min according to CLSI H21-A5 standards to isolate platelet-rich plasma, which was then analyzed immediately at room temperature. For Clauss fibrinogen, a 0.05 mL standard lyophilized titrated human calcium thrombin (approx. 80 NIH/mL standard solution), STA^®^ - Fibrinogen 5 (REF 00674), was diluted with 5 mL of distilled water according to the manufacturer’s direction and stored at 2–8°C for up to 14 days. The thrombin reagent solution was warmed to room temperature prior to assay. Mixture of plasma with the thrombin reagent was placed into the analyzer, which performs a 1:20 dilution with the manufacturer’s buffer solution STA^®^ - Owren-Koller (REF 00360) and converts the fibrinogen concentration to mg/dL based on a standard pre-calibrated curve. The reference range of fibrinogen concentration at our institution is 200–450 mg/dL. Platelet count was obtained using citrated whole blood on a Sysmex^®^ XN-9000 flow cytometer (Sysmex, Kobe, Japan).

### RAR Assays of Plasma Samples

Citrated frozen plasma samples obtained at the Henry Ford Hospital were brought to room temperature for 10 min for RAR assays. Each plasma sample was divided into nine equal portions for nine RAR reagent conditions that contain different concentrations of CaCl_2_ (Fluka), Rabbit Brain Cephalin (Lipids, Pel-Freez Biologicals), kaolin (Fisher Scientific), TF stock (10X Dade Innovin, Siemens), thrombin (Fisher Scientific) and 0.85% buffered saline (Fisher Scientific). The nine reagent conditions are: 1) Control (calcium 15 mM, lipids 25 μM); 2) CK (calcium 15 mM, lipids 25 μM and kaolin 0.004% w/w); 3) CRT (calcium 15 mM, lipids 25 μM, kaolin 2% w/w and TF 2 μg/mL); 4) TF 0.01% (calcium 15 mM, lipids 25 μM and TF 7 μg/mL); 5) TF 0.05% (calcium 15 mM, lipids 25 μM and TF 35 μg/mL); 6) TF 0.2% (calcium 15 mM, lipids 25 μM and TF 140 μg/mL); 7) thrombin 0.1 U/mL (calcium 15 mM, lipids 25 μM and thrombin 0.1 U/mL); 8) thrombin 0.2 U/mL (calcium 15 mM, lipids 25 μM and thrombin 0.2 U/mL); 9) thrombin 0.4 U/mL (calcium 15 mM, lipids 25 μM and thrombin 0.4 U/mL).

For each RAR assay, 90 μL of plasma sample was mixed with one of the nine reagents, and 100 μL of the mixture was immediately pipetted into one well in a 96-well plate for RAR, which started at 20 s after mixing.

### Measurement of Frequency of Resonant Surface Waves in a Sample using RAR

RAR measures a sample housed in a 96-well microplate using an excitation transducer (1.5 MHz) to generate resonant surface waves in the sample, which was detected by a detection transducer (7 MHz) operated in pulse-echo mode. A shift of the arrival time of the echo signal from the sample surface (Δt) from the pre-excitation signal was used to determine the surface displacement d=cΔt/2, where c is the speed of sound, to track the resonant surface waves. Fast Fourier Transform was performed to obtain the frequency spectrum of the resonant surface waves.

### Correlation of RAR parameters with Clinical Test Results

RAR parameters, Coagulation Start Time, Duration, and Final Resonant Frequency (FRF), were extracted from each RAR assay. Correlation coefficient of one RAR parameter with one clinical test result was calculated as

$$R^{\left(m,n\right)}=\frac{\sum_{i=1}^{38}\left(A_{i}^{\left(m\right)}-{\underline{A}}^{\left(m\right)}\right)\left(B_{i}^{\left(n\right)}-{\underline{B}}^{\left(n\right)}\right)}{\sqrt{\sum_{i=1}^{38}{\left(A_{i}^{\left(m\right)}-{\underline{A}}^{\left(m\right)}\right)}^{2}}\sqrt{\sum_{i=1}^{38}{\left(B_{i}^{\left(n\right)}-{\underline{B}}^{\left(n\right)}\right)}^{2}}}$$

where m=1 to 9, for RAR reagent conditions, n=1 to 16, representing clinical test results. For patient $i,\;A_{i}^{\left(m\right)}$ and $B_{i}^{\left(n\right)}$ are the RAR parameter for reagent condition m and clinical test result n, respectively, with ${\underline{A}}^{\left(m\right)}$ and ${\underline{B}}^{\left(n\right)}$ as the respective averages for the cohort of patients.

### Predicting Transfusion Requirements using a Quadratic Classifier

A two-class quadratic classifier (QC) considering the covariance/correlation among selected signal features was trained on RAR parameters and used as a prediction model to determine whether RAR parameters or clinical test results can correctly predict transfusion of fresh frozen plasma (FFP) and cryoprecipitate (CRYO). The QC model was also used to evaluate the prediction outcome of transfusion requirements of FFP and CRYO based on clinical test results.

## Figures and Tables

**Table 1:** Demographics summary of the 38 patients included in the study.

Age (median, IQR) (years)	56 (36–67)

**Female (n, %)**	10 (26.3%)

**Body Mass Index (median, IQR) (kg/m ^2^ )**	27.1 (25.2–34.2)

**Race (n, %)**	9 (23.7%)
African American	22 (57.9%)
Caucasian	2 (5.3%)
Other	5 (13.16%)
Unknown	

**Reason for hospitalization (n, %)**	5 (13.2%)
Cardiothoracic Surgery	5 (13.2%)
General Surgery	4 (10.5%)
Blunt Injury	1 (2.6%)
Penetrating Injury	1 (2.6%)
Heart Transplant	5 (13.2%)
Liver Transplant	1 (2.6%)
Kidney Transplant	10 (26.3%)
Decompensated Cirrhosis	1 (2.6%)
Septic Shock	1 (2.6%)
Post-cardiac arrest syndrome	2 (5.3%)
Cardiogenic shock on VA-ECMO	2 (5.3%)
Non-cirrhotic gastrointestinal bleed	

**Co-morbidities (n, %)**	13 (34.2%)
Cirrhosis	9 (23.7%)
Diabetes mellitus	22 (57.9%)
Hypertension	2 (5.3%)
Malignancy	9 (23.7%)
Heart Failure	6 (15.7%)
Coronary artery disease	13 (34.2%)
Chronic/end-stage kidney disease	5 (13.16%)
Unknown	

**Pre-admission anti-thrombotics (n, %)**	10 (26.3%)
Aspirin	3 (7.9%)
Warfarin	1 (2.6%)
Direct oral anticoagulant	2 (5.3%)
Unknown	

Expired as Inpatient (n, %)	14 (36.8%)

Abbreviations: IQR, interquartile range; VA-ECMO, venoarterial extracorporeal membrane oxygenation

**Table 2:** The nine RAR assay conditions with concentrations of various reagents.

Reagent Condition	Calcium (mM)	Lipid (μM)	Kaolin (% w/w)	TF (μM/mL)	Thrombin (Unit/mL)
#1: Control	15	25	-	-	-
#2: CK	15	25	0.004	-	-
#3: CRT	15	25	2	2	-
#4: TF 0.01%	15	25	-	7	-
#5: TF 0.05%	15	25	-	35	-
#6: TF 0.3%	15	25	-	140	-
#7: Thrombin 0.1 U/mL	15	25	-	-	0.1
#8: Thrombin 0.2 U/mL	15	25	-	-	0.2
#9: Thrombin 0.4 U/mL	15	25	-	-	0.4

Abbreviations: CK: citrated kaolin; CRT: citrated RapidTEG; TF: Tissue Factor

## Data Availability

The datasets generated during and/or analyzed during the current study are available from the corresponding author upon reasonable request.
